# Targeted blockade of TGF-β and IL-6/JAK2/STAT3 pathways inhibits lung cancer growth promoted by bone marrow-derived myofibroblasts

**DOI:** 10.1038/s41598-017-09020-8

**Published:** 2017-08-17

**Authors:** Jindong Shi, Jingjing Feng, Juan Xie, Zhoufang Mei, Tianyun Shi, Shengmei Wang, Yong Du, Gong Yang, Yougen Wu, Xiaojiao Cheng, Shanqun Li, Liming Zhu, Chung S. Yang, Shuiping Tu, Zhijun Jie

**Affiliations:** 1 Department of Respiratory Medicine, The Fifth People’s Hospital of Shanghai, Fudan University, Shanghai, 200240 China; 2 Department of Central Laboratory, The Fifth People’s Hospital of Shanghai, Fudan University, Shanghai, 200240 China; 3 Department of Gastroenterology, Ruijin Hospital, Shanghai Jiaotong University School of Medicine, Shanghai, 200025 China; 4 Department of Gastroenterology, Ruijin Hospital North, Shanghai Jiaotong University School of Medicine, Shanghai, 201821 China; 5 Departments of Respiratory Medicine, Zhongshan Hospital, Fudan University, Shanghai, 200032 China; 6 Department of Chemical Biology, Ernest Mario School of Pharmacy, Rutgers, The State University of New Jersey, Piscataway, NJ 08854 USA

## Abstract

To investigate the role of TGF-β and IL-6 in myofibroblasts (MFs) — lung cancer cell interactions, lung cancer cells (Lewis and CTM-167 cell lines) were stimulated by IL-6, MF-conditioned medium (MF-CM) or MFs, with or without TGF-β signaling inhibitor — SB431542 and/or JAK2/STAT3 inhibitor — JSI-124. MFs were stimulated by TGF-β, cancer cell-CM or cancer cells, with or without SB431542 and JSI-124. Cell proliferation, the levels of cytokines, expression of mRNA and protein were determined. Mice bearing xenograft tumors were intraperitoneally treated with SB431542 or JSI-124 and monitored for up to 45 days. In co-culture systems, MFs secreted high levels of IL-6, while cancer cells produced high levels of TGF-β. Recombinant IL-6 and MF-CM activated STAT3 and upregulated TGF-β in cancer cells. In contrast, cancer cell-CM or TGF-β stimulated MFs to produce IL-6. Blockade of JAK2/STAT3 and TGF-β signaling by specific inhibitors significantly inhibited cell proliferation *in vitro* and tumor growth *in vivo* of lung cancer cells. Our study demontrated that the TGF-β and IL-6/JAK2/STAT3 signaling pathways form a positive feedback signaling loop that mediated the interactions between MFs and lung cancer cells. Targeted inhibiton of this signaling loop could be a new approach for lung cancer prevention and therapy.

## Introduction

Lung cancer is a primary cause of cancer-associated morbidity and mortality, with over 1 million deaths from the disease worldwide per year^1^. Within the spectrum of functional deficits associated with lung cancer are breathing disorders such as chronic pulmonary obstructive disease, emphysema, and asthma, which further reduces the quality of life for patients. A commonality between these associated functional pathologies is a connective tissue dysregulation at least in part mediated by inflammatory events that occur within the lung tissue and tumor stroma^2^. It is within the tumor microenvironment that myoblasts are linked to the progression of lung cancer. Although the involvement of myofibroblasts (MFs) in these tumor microenvironment has been studied and their contribution to tumor progression have been elucidated, the exact role of MFs in these complicated signaling cascades remains unclear.

It is known that the interaction between MFs and lung cancer cells influences the formation and progression of lung cancer development^3–5^. The roles of cytokines and other paracrine factors in such interactions are just beginning to be uncovered. Interleukin-6 (IL-6) is a dynamic cytokine that is known to play a role in immune responses and inflammation, as well as in various epithelial tumors^6^. IL-6, upon binding to its receptor, activates intracellular signaling through JAK tyrosine kinases and is amplified by other downstream signaling effectors including PI3K, MAPKs, and STATs^7^. Changes in signaling via another pro-inflammatory cytokine, transforming growth factor-β (TGF-β), are also closely linked to various activities related to cancer onset and migration^8–10^. Signaling pathways regulated by TGF-β in lung cancer cells include Wnt/β-catenin, MAPK, and JAK/STAT3 signaling^11^.

Recent studies indicate that patients with diagnosed lung cancer have elevated serum IL-6 (compared with normal subjects) and it is correlated with poor prognosis^12, 13^. Given the stimulatory role of IL-6 on JAK/STAT signaling, IL-6/JAK/STAT3 signaling may be involved in lung cancer progression. Epithelial-mesenchymal transition occurs when epithelial cells take on mesenchymal properties and is important for progression and metastasis of cancer. As such, pro-inflammatory cytokine signaling through JAK/STAT3 could be critical for the interactions between lung cancer cells and stromal cells in the transformation of lung cancer cells to take on stromal cell properties, which promote progression of lung cancer and result in poor prognosis^14^.

Gaining a better understanding of the inflammatory signaling cascades in the interaction between lung cancer cells and MFs would aid in the development of approaches for inhibiting cancer progression. The present study aims to investigate the roles that TGF-β and IL-6/JAK2/STAT3 signaling play in cancer cell-MF interaction and how this interaction could influence cancer cell proliferation and disease progression in both *in vitro* and *in vivo* systems.

## Results

### Lung cancer cell-produced TGF-β induces MF proliferation and cytokine secretion

**Figure 1:**
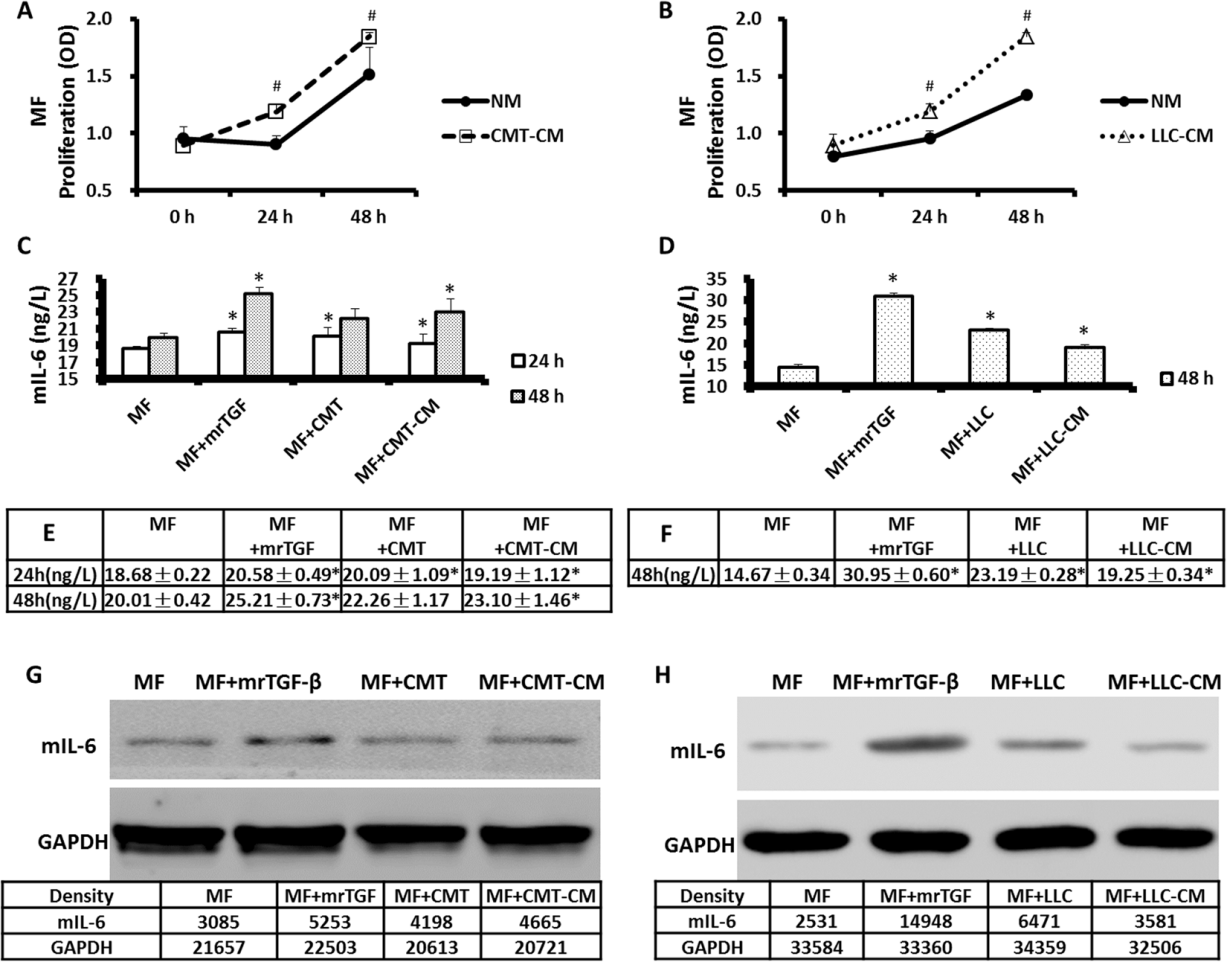
Lung cancer cells promoted MFs proliferation and cytokine secretion via TGF-β. MFs were cultured in normal medium, CMT-167-conditioned medium (CMT-CM) or LLC-conditioned medium (LLC-CM) and the proliferation of MFs was measured by CCK-8 at 0 h, 24 h and 48 h (**A**,**B**). Cancer-CM enhanced MF proliferation. In a second set of experiments, MFs were cultured alone, with mrTGF-β (2 ng/mL), with caner condition-medium or co-cultured with CMT/LLC cells. mIL-6 concentration in supernatant was measured by ELISA (**C**–**F**) and mIL-6 protein in MFs was assessed by Western blots (**G**,**H**). mrTGF-β, lung cancer cells and cancer-CM enhanced expression of mIL-6 in MFs. n = 6 wells/group; *Compared to MF group, p < 0.05; ^#^Compared to NM group, p < 0.05.

To measure MF proliferation, we cultured the cells with different treatments, and CCK-8 was assayed at 0 h, 24 h and 48 h. Although normal lung cancer cell culture medium stimulated MF proliferation over time, culture in Mouse non-small lung cancer cell line-conditioned medium (CMT-167-CM) and Lewis lung cancer cell line-conditioned medium (LLC–CM) further enhanced MF cell proliferation as determined through CCK-8 release (Fig. 1A and B). At both 24 h and 48 h, in addition of mrTGF-β (mouse recombinant TGF-β), co-cultureing with CMT-167 cells, and culturing with CMT-167-CM increased all mIL-6 protein levels (Fig. 1C and E) and at 48 h, co-cultureing with LLC cells, and culturing with LLC-CM (Fig. 1D and F) both elevated IL-6 production by MFs in comparison to MFs culture alone (*p* < 0.05). mRNA levels in MFs under these different culture conditions were similar at both 24 h and 48 h (Supplemental Fig. 1A,B). Western blotting results for mIL-6 protein were consistent with the ELISA data showing that mIL-6 expression was increased by all treatments over normal culture conditions, especially mrTGF-β (Fig. 1G and H).

### MFs promote lung cancer cell proliferation and cytokine production via IL-6

**Figure 2:**
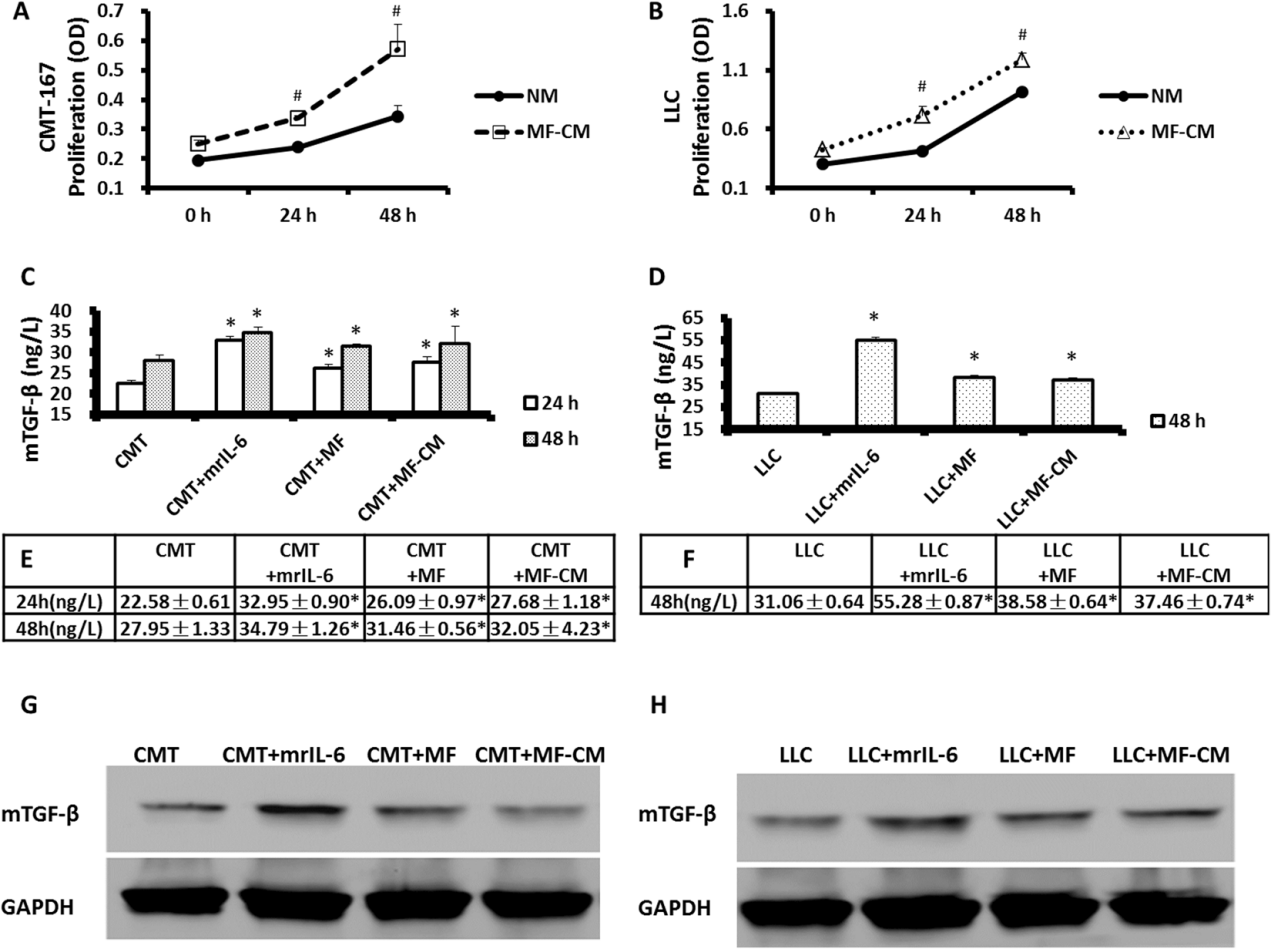
MFs promoted lung cancer cell proliferation and cytokine production via IL-6. CMT/LLC cells were cultured in normal medium or MF-CM and the proliferation of CMT/LLC cells was measured by CCK-8 at 0 h, 24 h and 48 h (**A**,**B**). MF-CM was able to promote cancer cell proliferation. CMT/LLC cells were cultured alone, with mrIL-6 (20 ng/mL) or with MF-CM, or co-cultured with MFs. mTGF-β concentration in supernatant was measured by ELISA (**C**–**F**). mTGF-β protein levels were measured by Western blots (**G**,**H**). mrIL-6, MFs and MF-CM were both able to enhance the mTGF-β expression in lung cancer cells. n = 6 wells/group; *Compared to CMT or LLC group, *p* < 0.05; ^#^Compared to NM group, *p* < 0.05.

Cancer cell lines (CMT/LLC) were cultured in normal medium or MF-CM and their proliferation was assessed by CCK-8 at 0 h, 24 h and 48 h (Fig. 2A and B). The results showed that treatment with MF-CM promoted the proliferation of cancer cells.

To determine if IL-6 was promoting this proliferation and TGF-β expression by cancer cells, CMT/LLC cells were cultured in 6-well plates by themselves in normal medium or treated with mrIL-6 (mouse recombinant IL-6), or co-cultured with MFs in a transwell system. mTGF-β concentrations in supernatant were measured by ELISA (Fig. 2C–F). Related mTGF-β protein levels and mRNA levels by CMT/LLC cells were measured by Western blots (Fig. 2G and H) and by RT-PCR (Supplemental Fig. 2A,B). Our results showed that mrIL-6, MFs and MF-CM significantly enhanced the expression of mTGF-β in these lung cancer cells (*p < 0.05 compared with the MF alone group).

### Inhibitors of TGF-β and JAK2/STAT3 prevent MF and CMT/LLC cell proliferation in a co-culture system

To analyze the signaling pathways involved in MF and cancer cell proliferation, MFs or CMT/LLC cells were cultured in normal or conditioned medium. Proliferation of MFs and CMT/LLC cells was measured by CCK-8 at 0 h, 24 h and 48 h.

**Figure 3:**
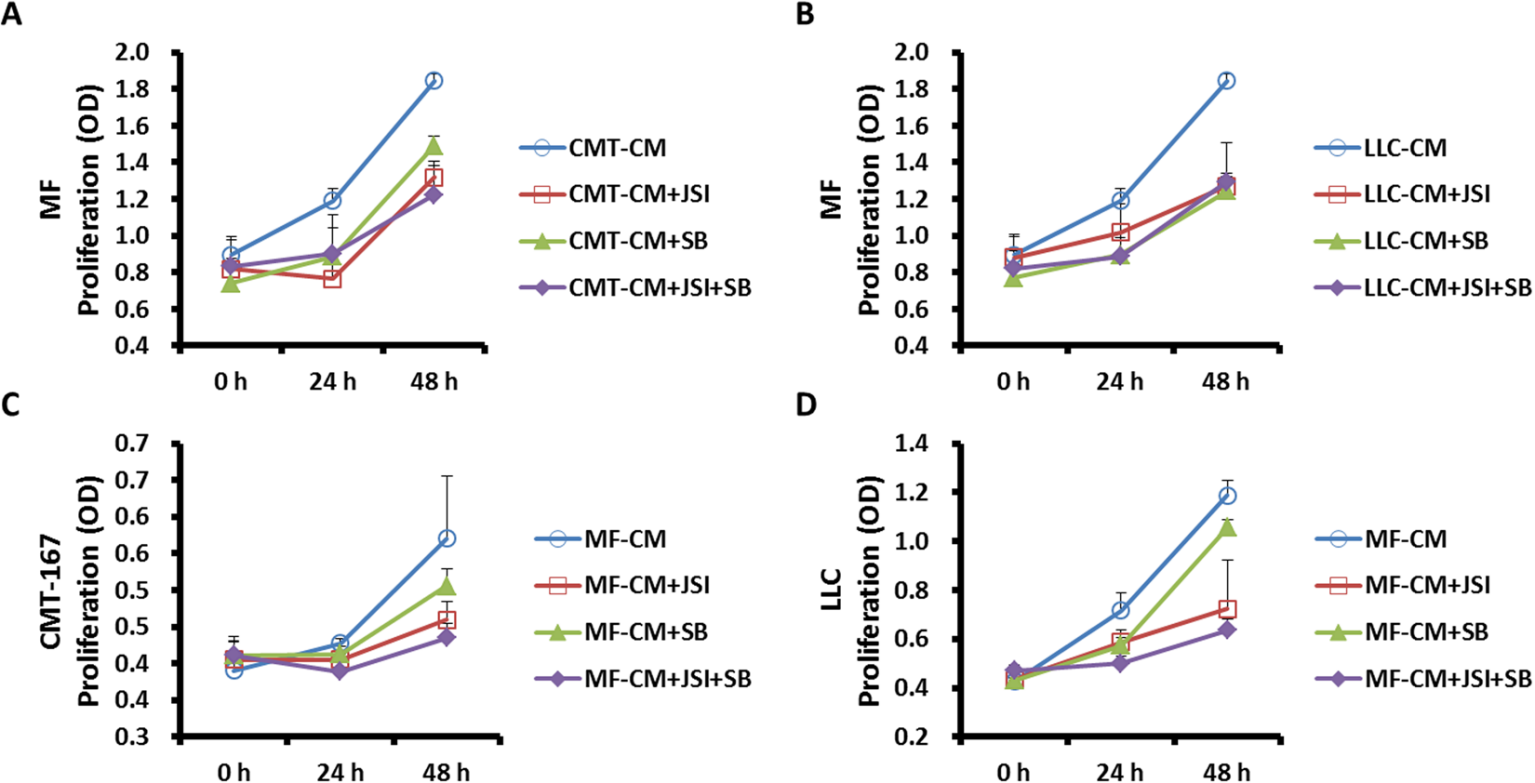
SB-431542 and JSI-124 inhibited the proliferation of MFs and CMT/LLC cells in co-culture system. MFs or CMT/LLC cells were cultured in conditioned medium. Cell proliferation was measured by CCK-8 at 0 h, 24 h and 48 h. SB-431542 (10 μM), JSI-124 (0.2 μM), or SB-431542 (5 μM) + JSI-124 (0.1 μM) inhibited MF proliferation in CMT/LLC-CM (**A**,**B**). SB-431542 (10 μM), JSI-124 (0.2 μM), or SB-431542 (5 μM) + JSI-124 (0.1 μM) inhibited CMT/LLC cell proliferation in MF-CM (**C**,**D**). n = 6 wells/group.

Application of the inhibitors of TGF-β receptor kinase — SB-431542 (10 μM), inhibitor of JAK2/STAT3 signaling — JSI-124 (0.2 μM), or a combination of the two (5 μM SB-431542 + 0.1 μM JSI-124) all showed inhibitory effects on MF proliferation when cultured in CMT/LLC-CM (Fig. 3A and B) and in normal medium (Supplemental Fig. 3A). Likewise, both inhibitors and their combinations inhibited CMT/LLC cell proliferation when cultured in MF-CM (Fig. 3C and D) and in normal medium (Supplemental Fig. 3B,C).

### Inhibitors of TGF-β and JAK2/STAT3 block production of IL-6 by MFs and TGF-β by lung cancers, respectively

**Figure 4:**
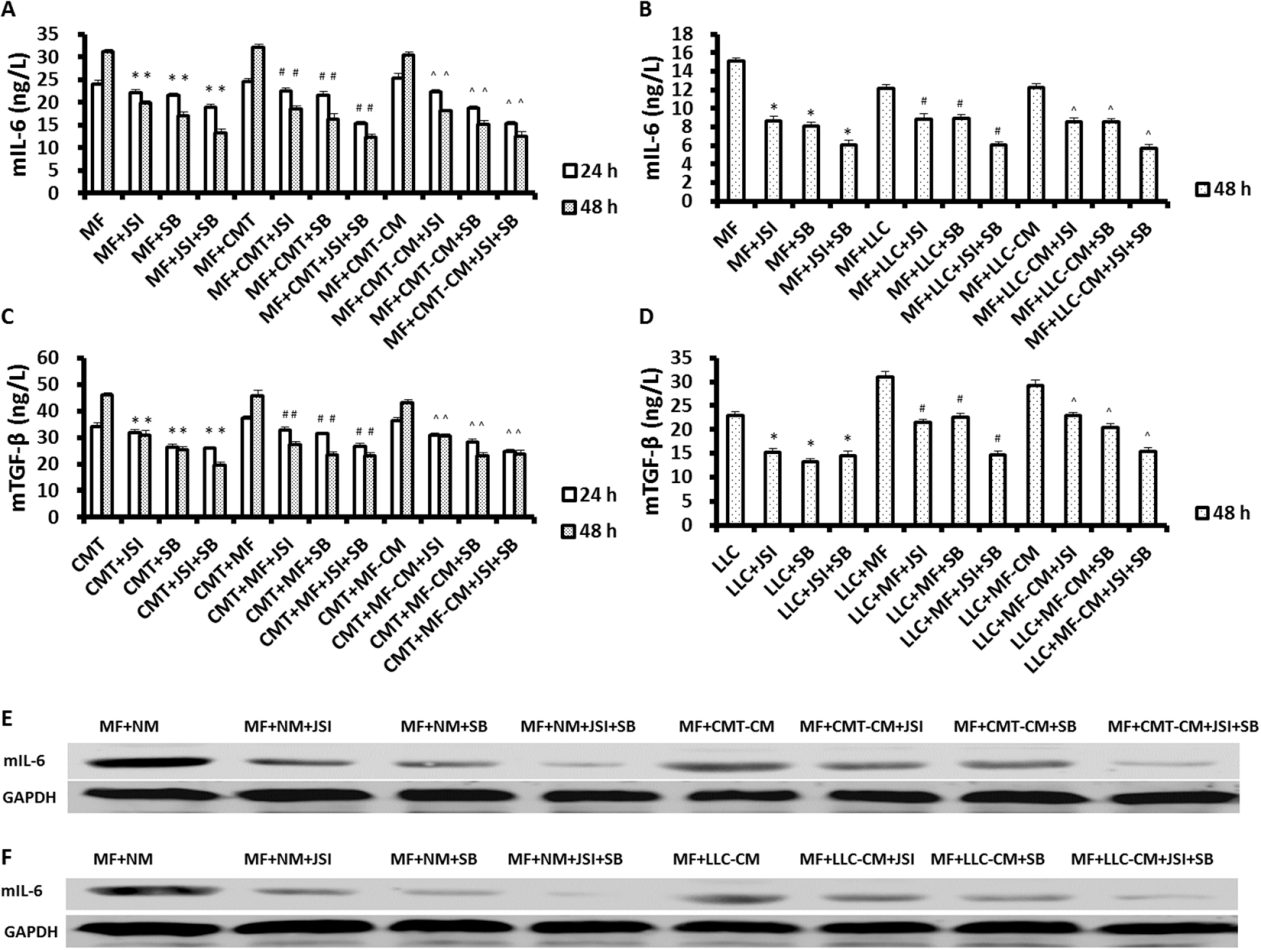
SB-431542 and JSI-124 inhibit production of IL-6 by MFs and production of TGF-β by lung cancer cells. MFs (5 × 10^4^/well) or CMT/LLC cells (5 × 10^4^/well) were cultured in 6-well plates. In co-culturing, CMT/LLC cells were loaded on the upper chamber and MFs were loaded on the lower chamber of a transwell system. SB-431542 (10 μM), JSI-124 (0.2 μM), or SB-431542 (5 μM) + JSI-124 (0.1 μM) blocked mIL-6 protein production by MFs and mTGF-β protein production by CMT/LLC cells in normal and conditioned medium, as determined by ELISA (**A**–**D**) and by Western Blots (**E**,**F**). n = 6 wells/group; *Compared to MF or cancer cell group, *p* < 0.05; ^#^Compared to MF + cancer cell co-culturing group, *p* < 0.05; ^^^Compared to MF or cancer cell culturing in conditioned medium group, *p* < 0.05.

MFs and/or CMT/LLC cells were cultured separately, or together in a transwell culture system. These cells were cultured in MF-CM or CMT/LLC-CM. To assess the effects of TGF-β and related signaling pathways on growth factor and cytokine secretion, the cell groups were treated with the inhibitors of SB-431542 (10 μM), JSI-124 (0.2 μM), or a combination of the two (5 μM SB-431542 + 0.1 μM JSI-124). SB-431542, JSI-124, and the combination significantly inhibited mIL-6 protein production by MFs and mTGF-β protein production by CMT/LLC cells in normal medium and conditioned medium as assayed by ELISA (Fig. 4A–D), and by Western Blots (Fig. 4E and F). mIL-6 mRNA expression of MFs and mTGF-β mRNA expression of CMT/LLC cells were inhibited by SB-431542, JSI-124, and a combination of the two (Supplemental Fig. 4A–D).

### Inhibitors of TGF-β and JAK2/STAT3 reduce JAK2, STAT3, survivin and c-myc gene expression in lung cancer cells

**Figure 5:**
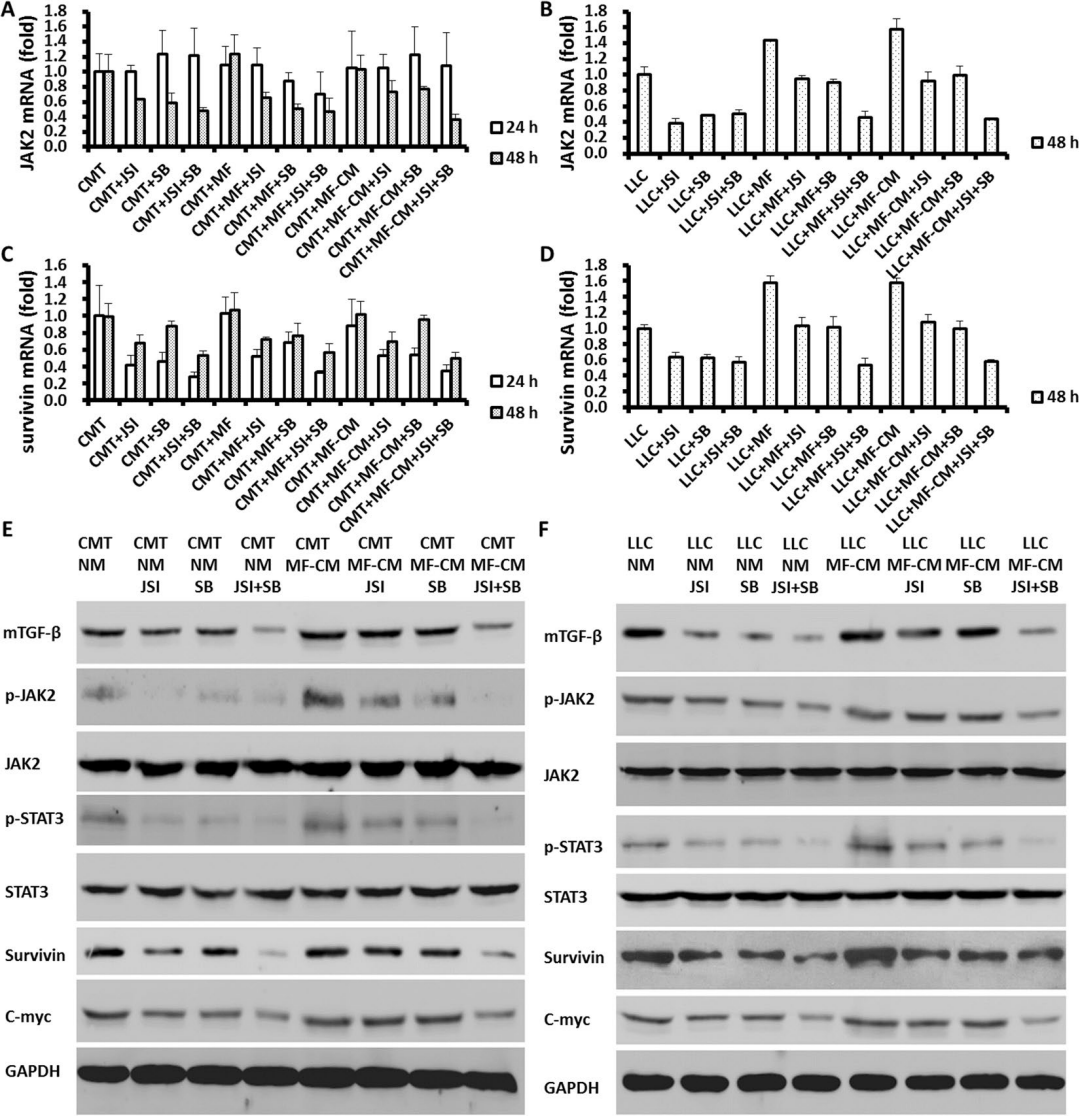
SB431245 and JSI-124 decreased the gene expression of JAK2, STAT3, survivin and c-myc in lung cancer cells. CMT/LLC cells were cultured in 6-well plates. CMT/LLC cells and MFs were co-cultured in transwell plates. SB-431542 (10 μM), JSI-124 (0.2 μM), or SB-431542 (5 μM) + JSI-124 (0.1 μM) inhibited expression of JAK2, STAT3, survivin and c-myc genes in lung cancer cells (**A**–**D**) in normal medium and MF-CM, as determined by Western Blots (**E**,**F**). n = 6 wells/group.

CMT/LLC cells (5 × 10^4^/well) were cultured in 6-well plates for single culture experiments. CMT/LLC cells (5 × 10^4^/well) and MFs (5 × 10^4^/well) were loaded in the upper and lower chambers, respectively, of a transwell system. Treatment with SB-431542 (10 μM), JSI-124 (0.2 μM), or SB-431542 (5 μM) + JSI-124 (0.1 μM) inhibited the expression of JAK2, STAT3, survivin and c-myc in lung cancer cells in normal medium and MF-CM, based on RT-PCR measurements (Fig. 5A–D and Supplemental Fig. 5A–D) and by Western Blots (Fig. 5E and F).

### Inhibitors of TGF-β and IL-6/JAK2/STAT3 signaling pathways inhibit lung cancer growth promoted by MFs *in vivo*

**Figure 6:**
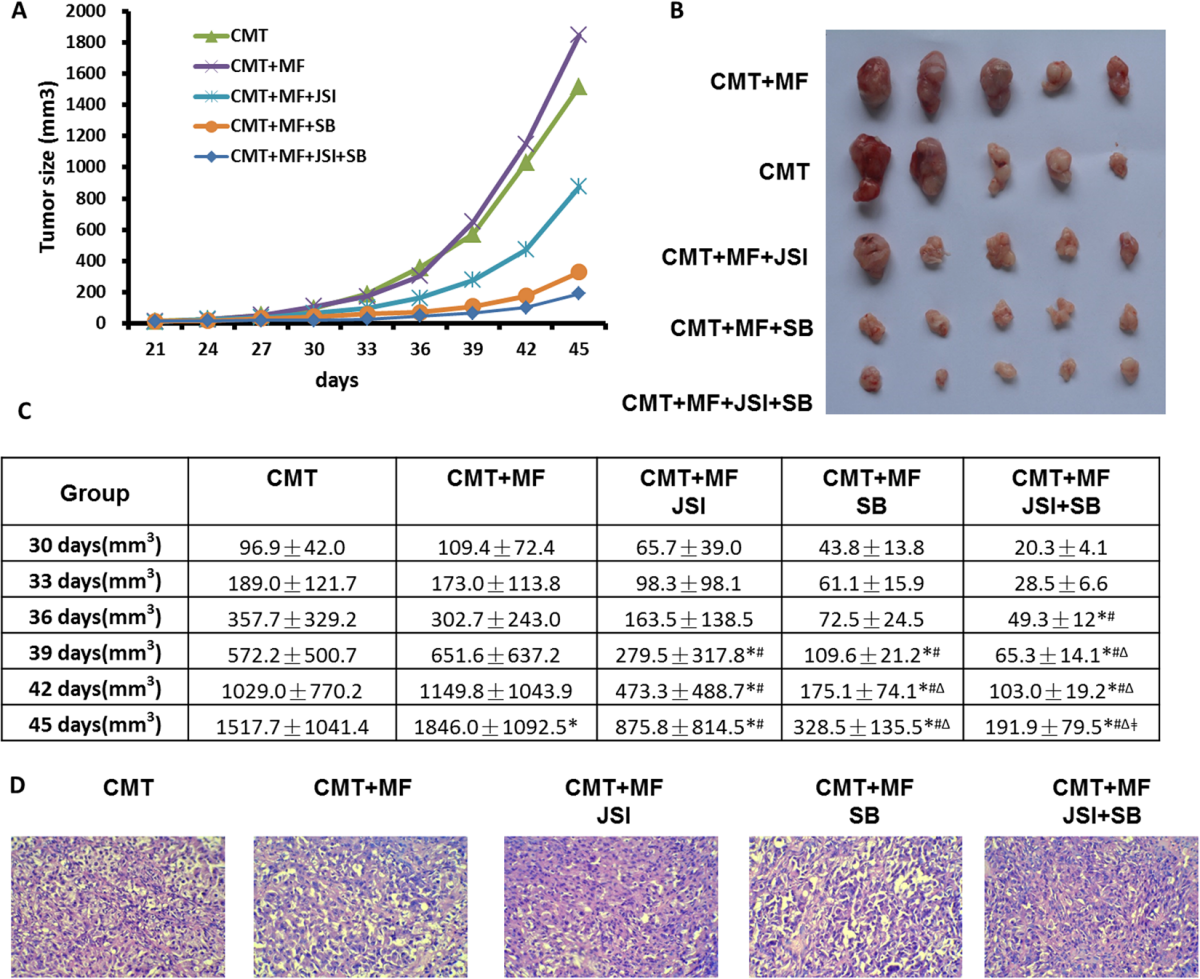
Inhibitors of TGF-β and IL-6/JAK2/STAT3 signaling pathways retarded lung cancer growth promoted by MFs *in vivo*. Six-week old BALB/c athymic nude mice (n = 5 mice/group) were injected *s*.*c*. with CMT-167 cells (2 × 10^6^/mouse), or CMT-167 cells (2 × 10^6^) + MFs (2 × 10^6^) per mouse. SB-431542 (10 mg/kg body weight), JSI-124 (0.5 mg/kg body weight), or SB-431542 (5 mg/kg body weight) + JSI-124 (0.25 mg/kg body weight) were administered in 20% DMSO intraperitoneal three times per week starting one day after tumor cell inoculation. Tumor size was measured every 3 days (**A**–**C**). Mice were sacrificed on 45 days and H&E stained tumor tissue slides are shown (**D**). ^*^Compared to CMT group, *p* < 0.05; ^#^Compared to CMT+MF group, *p* < 0.05; ^Δ^Compared to CMT+MF+JSI group, *p* < 0.05; ^ǂ^Compared to CMT+MF+SB group, *p* < 0.05.

To assess the role of TGF-β and IL-6/JAK2/STAT3 signaling in tumor formation *in vivo*, six-week-old BALB/c athymic nude mice (n = 5 mice/group) were injected subcutaneously with CMT-167 (2 × 10^6^/mouse), or CMT-167 (2 × 10^6^) + MFs (2 × 10^6^) per mouse. The inhibitors, SB-431542, JSI-124, or SB-431542 + JSI-124 all reduced tumor size over time, with the combined inhibitor treatment showing the most pronounced effect in tumor size reduction (Fig. 6A–D).

### Treatment with SB-431542 and JSI-124 reduces cytokine expression in mouse tumor tissues

**Figure 7:**
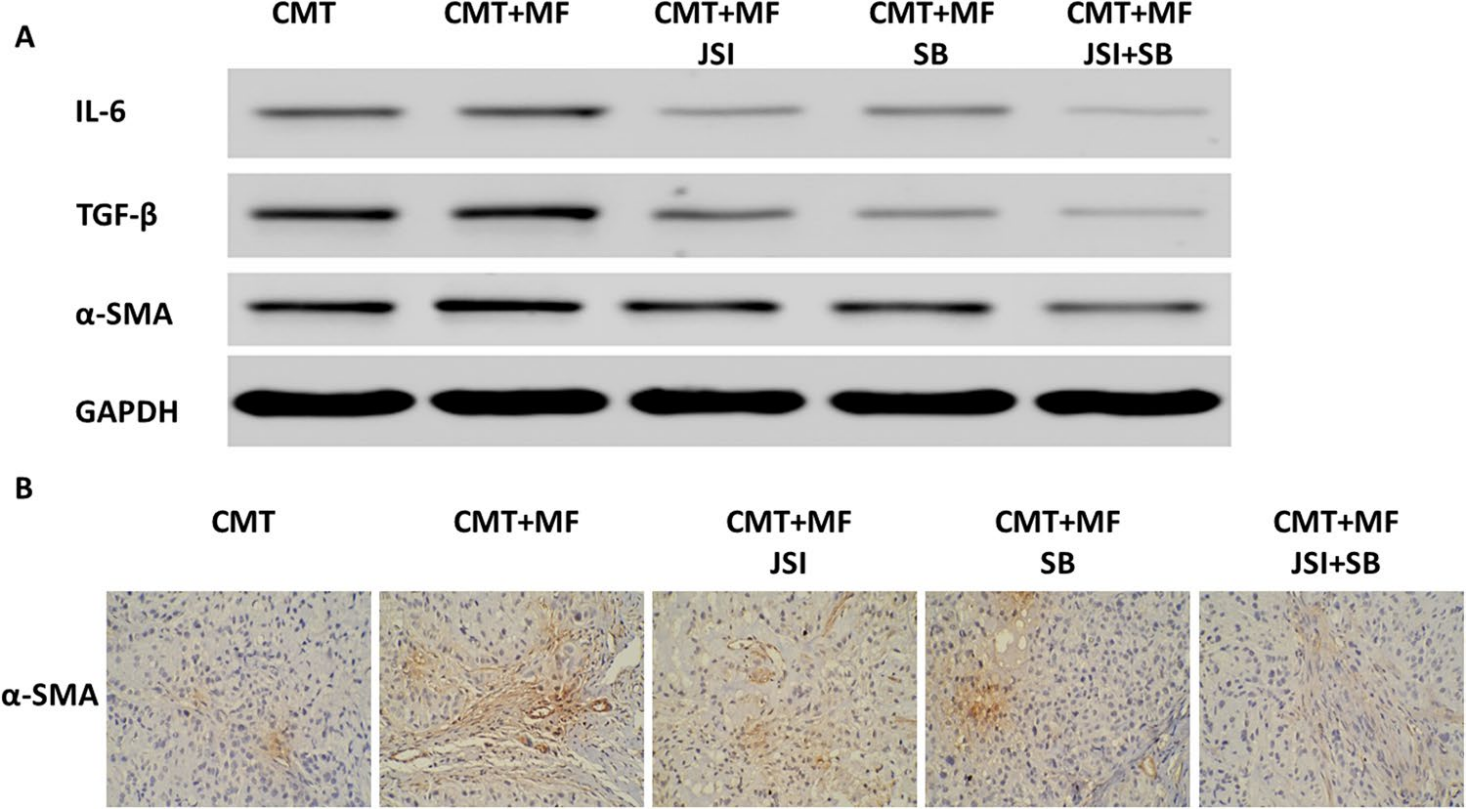
Effects of SB-431542 and JSI-124 on cytokine expression in tumor tissues of mice. The protein expression of IL-6, TGF-β and α-SMA in tumor tissues of mice was determined by Western Blots (**A**). α-SMA expression in tumor tissues was also analyzed by immunohistochemistry (Magnification 200×) (**B**).

The protein expression of IL-6, TGF-β and α-SMA in tumor tissues from BALB/c athymic nude mouse was determined by Western Blots (Fig. 7A). α-SMA expression in tumor tissues was also analyzed by immunohistochemistry (Fig. 7B). Based on the blots, it appeared that each inhibitor and the combination of inhibitors reduced expression of IL-6, TGF-β and α-SMA protein in tumor tissues isolated from the mice injected with CMT-167, MF and CMT + MF. Likewise, the immunohistochemical staining of α-SMA was reduced in histological sections of tumor tissue in each of the inhibitor-treated mouse group.

## Discussion

In the present study, we aimed to elucidate the mechanisms by which MFs and cancer cell interact to produce cytokines and growth factors *in vitro* and *in vivo*. We hope these studies will shed light on mechanisms that may be targeted to inhibit tumorigenesis, tumor progrsssion and metastasis of lung cancers in a clinical setting. These studies build upon important previous findings that highlight key signaling pathways, such as JAK/STAT and TGF-β in the interaction between lung cancer cells and MFs.

Tumor tissues are not simply made up of cancer cells, but also other cells that comprise vasculature, inflammatory cells, and fibroblasts. The interaction between cancer cells and the stromal microenvironment is crucial for tumor progression and metastasis. Of the stromal tissue involved in this process, MFs are well documented to promote cancer develoment and progression^15–17^ and are considered the primary stromal cell type within human tumors^18^. MFs also contribute to migratory action of cancer cells and tumor angiogensis^19, 20^. The interplay between MFs and cancer cells is mediated by contact, as well as by paracrine-mediated signaling between the cell types. It is generally accepted that MFs are in contact with cancer cells, and express α-SMA^21^. The process through which stromal fibroblasts differentiate into MFs and contribute to cancer progression is of great interest, and this process may be targeted for cancer theraputics.

TGF-β is thought to be a key paracrine factor that triggers differentiation of MFs by cancer cells^22, 23^. Similarly, TGF-β is also likely to trigger a paracrine feedback loop by which differentiated stromal-origin MFs promote cancer-supporting processes and events^22^. Our findings support the role of TGF-β in enhancing proliferation of MFs. When MFs were cultured with cancer cell-CM, proliferation of MFs increased. Importantly, by inhibiting TGF-β signaling, we were able to prevent the effect of cancer-CM on proliferation of MFs. We also found that cancer cells and cancer-CM promoted IL-6 secretion by MFs.

IL-6 has been previously shown as a growth-promoting factor for cancer cells^24^. We showed that MFs secrete IL-6 in response to factors secreted by cancer cells. MFs and MF-CM induced cancer cell proliferation, similar to the action of IL-6. Therefore, TGF-β and IL-6 form an important paracrine signaling cycle between MFs and cancer cells, and this signal cycle contributes to cancer progression.

Our studies with inhibitors of TGF-β and JAK/STAT3 signaling implicate these pathways as a key mechanism by which TGF-β influences growth and cytokine expression between these two cell types. Blockade of TGF-β as well as JAK2/STAT3 signaling reduced the proliferation and cytokine production of both cancer cells and MFs in culture (Figs 3 and 5). This is consistent with previous observations that IL-6 activates cancer cell growth^25^ and prevents cancer cell death via STAT3 activation^26, 27^. IL-6-mediated STAT3 activation has been shown to promote drug resistance in cancer cells^27^, underscoring the importance of such cytokine signaling between stromal MFs and cancer cells as a possible target for cancer therapeutic development.

To further support our *in vitro* result, we inoculated athymic nude mice with MFs and cancer cells, and found that suppression of TGF-β and JAK2/STAT3 signaling by inhibitors reduced tumor size and minimized tumor histopathology *in vivo* (Fig. 6). We also observed that inhibiting these signaling pathways reduced α-SMA expression in tumor tissues. There is evidence that α-SMA-negative MFs do not promote cancer cell-associated behaviors that are found in α-SMA-positive MFs^20, 22^. As such, our study extends findings concerning mechanisms of paracrine-mediated interactions between MFs and cancer cells in an animal model, and provides support for the use of these cytokines as targets for preventing cancer progression.

Future studies should explore the role of MF differentiation and its involvement in tumor formation and progression. Immune cell infiltration, such as by CD_8_^+^ T-lymphocytes, is an important immune response to cancer cells and positively correlated with good prognosis in various cancers^28–31^. Some observations suggest that MFs may prevent interaction between such infiltrating immune cells with cancer cells, thus preventing immune-mediated anti-cancer responses^32^. This mechanism, coupled with the influence of cancer-associated fibroblasts with mechanical and matrix remodel influences on the tumor microenvironment^33^, could be important topics for future studies.

In conclusion, our study expands upon prior research in providing evidence for an important paracrine-mediated interaction between cancer cells and stromal-origin MFs in tumor formation and progression. Our findings highlight mechanistic importance of TGF-β and JAK2/STAT3 signaling in this positive feedback loop *in vitro* and *in vivo*. Future studies will further investigate specific mechanisms and inhibitors for the interactions between these two cell types to aid in the development of effective cancer therapeutics.

## Materials and Methods

### Cell culture and reagents

CMT-167 cells and LLC cells were purchased from the Cell Bank of the Chinese Academy of Sciences (Shanghai, China). Bone marrow-derived myofibroblasts (MFs) were isolated in our laboratory from dysplastic gastric tissues of EGFP^+^ bone marrow-transplanted IL-1β transgenic mice based on previous methods^34^. RPMI 1640 medium containing 10% FBS, 100 U/mL penicillin, and 100 μg/mL streptomycin (Gibco BRL, Grand Island, NY, USA). Cucurbitacin I (JSI-124) and SB 431542 were purchased from Sigma (St. Louis, MO, USA) and were dissolved in dimethyl sulfoxide (DMSO; Sigma, St. Louis, USA) and stored at −20 °C. Mouse recombinant IL-6 (mrIL-6) and mouse recombinant TGF-β (mrTGF-β) were purchased from RD (Minneapolis, MN, USA).

For the production and isolation of MF conditioned medium (MF-CM) and lung cancer cell conditioned medium (CMT-CM and LLC-CM), MFs, CMT and LLC cells were cultured in RPMI 1640 medium containing 10% fetal bovine serum (FBS), 100 U/mL penicillin, and 100 μg/mL streptomycin. Culture medium was changed once cells were 70% confluent in 10 cm dishes. The final medium for experimental use was collected after 24 h and centrifuged at 800 rpm for 5 min. The supernatant was passed through a 0.45 μm filter to remove any remaining debris and cell components and stored at 4 °C. In experiments involving cell transplantation for tumor formation in mice, cells were passed through a 40 μm filter for isolation. An enzyme-linked immunosorbent assay (ELISA) kit was purchased from BD Biosciences (San Jose, CA, USA) for cell and tissue protein quantification. For Western blotting, gels and reagents were purchased from Millipore (Bedford, MA, USA). Absorbance was measured at 450 nm by a Multiscan MC reader (Labsystems Multiskan, MS, Finland).

### Transcription reverse-polymerase chain reaction

Total RNA was extracted from cultured cells with Trizol reagent (Invitrogen, Carlsbad, CA). RNA concentration was measured with an ABI-7300 analyzer (Applied Biosystems ABI, USA). Complementary deoxyribonucleic acid (cDNA) was synthesized using a reverse transcription kit (Thermo Fisher Scientific, Beijing, China), and real-time polymerase chain reaction (RT-PCR) was performed with SYBR green (Thermo Fisher Scientific, Beijing, China). Mouse primer sequences used were as follows (forward and reverse primers):

**mIL-6** Forward primer 5′-CTGCAAGTGCATCATCGTTGTT-3′, Reverse primer 5′-CCGGAGAGGAGACTTCACAGAG-3′;

**mTGF-β** Forward primer 5′-AAGGACCTGGGTTGGAAGTG-3′, Reverse primer 5′-TGGTTGTAGAGGGCAAGGAC-3′;

**JAK2** Forward primer 5′-GCAGCAGCAGAACCTACAG-3′, Reverse primer 5′-CTAACACCGCCATCCCAAG-3′;

**STAT3** Forward primer 5′-GACTCAAAGCCACCTCATTC-3′, Reverse primer 5′-GCCTTGCCTTCCTAAATACC-3′;

**Survivin** Forward primer 5′-CCTTCGTGTCTGTTCATCATTC-3′, Reverse primer 5′-TGTCACTCAGGTCCAAGTTATC-3′

**c-myc** Forward primer 5′-TGAGGAAACGACGAGAACAG-3′, Reverse primer 5′-AGCCAAGGTTGTGAGGTTAG-3′

**GAPDH** Forward primer 5′-ATCACTGCCACCCAGAAG-3′, Reverse primer 5′-TCCACGACGGACACATTG-3′.

### Paracrine effects of cancer cells and MFs on cellular proliferation

To determine paracrine cellular affects between cancer cells and MFs, CMT/LLC cells (2 × 10^3^/well) or MFs (2 × 10^3^/well) were cultured in normal medium (NM) in 96-well plates. After 24 h changed the medium to NM, MF-CM or CMT/LLC-CM and the proliferation of CMT/LLC cells or MFs were measured by CCK-8 at 0 h, 24 h and 48 h. To assess cell proliferation, a CCK-8 kit was purchased from Dojindo Chemical Co., Japan. Proliferation was quantified as optical density following assay.

### Paracrine effects of cancer cells and MFs on TGF-β and IL-6 protein and gene expression, and effects of cytokines on cell proliferation

CMT/LLC cells (5 × 10^4^/well) and MFs (5 × 10^4^/well) with 2 ml RPMI 1640 were loaded into 6-well plates in CMT/LLC + MF group. CMT/LLC cells (5 × 10^4^/well) with 1 ml RPMI 1640 were loaded in lower side of transwell and MFs (5 × 10^4^/well) with 1 ml RPMI 1640 were loaded in upper side of 6-well transwell plate in the MF + CMT/LLC-CM group. mTGF-β and mIL-6 concentrations in the supernatants were measured by ELISA, mRNA levels of CMT/LLC cells were measured by RT-PCR. To assess the effects of the specific cytokine effects on the cells, CMT/LLC cells (5 × 10^4^/well) were cultured in 6-well plates alone or with mrIL-6 (20 ng/mL) or mrTGF-β (2 ng/mL).

### *In vitro* inhibition of TGF-β and JAK2/STAT3 signaling

To inhibit signaling pathways, CMT/LLC cells (5 × 10^4^/well) or MFs (5 × 10^4^/well) with 2 ml RPMI 1640 were loaded in 6-well plates for culture, and also together in a CMT/LLC + MF with 2 ml RPMI 1640 mixed culture group. For mixed culture, CMT/LLC cells (5 × 10^4^/well) with 1 ml RPMI 1640 and MFs (5 × 10^4^/well) with 1 ml RPMI 1640 were loaded in the lower and upper chambers of a transwell plate in MF-CM groups. To inhibit TGF-β and JAK2/STAT3 signaling, SB-431542 (10 μM), JSI-124 (0.2 μM), or SB-431542 (5 μM) + JSI-124 (0.1 μM) were used, and the gene expression and protein of JAK2, STAT3, survivin and c-myc in lung cancer cells were determined by RT-PCR and Western Blots.

### Tumor xenograft models and treatments

Six-week old male BALB/c athymic nude mice (Shanghai Slac laboratory animal Co., Ltd., Shanghai, China) were divided into 5 groups (n = 5 mice/group). All animal protocols were approved by the Animal Care and Facilities Committee of Fudan University. CMT-167 cells (2 × 10^6^)/mouse, or CMT-167 cells (2 × 10^6^) + MFs (2 × 10^6^)/mouse were injected subcutaneously (*s*.*c*.). For *in vivo* inhibitor treatment, SB-431542 (10 mg/kg body weight in 20% DMSO), JSI-124 (0.5 mg/kg body weight), or SB-431542 (5 mg/kg body weight in 20% DMSO) + JSI-124 (0.25 mg/kg body weight) were filter-sterilised and administered intraperitoneally three times per week starting one day after tumor cell inoculation, and control mice receive the same volume of diluent. All mice were monitored daily and euthanized after 45 days. Tumors were processed for further histology analysis, immunohistochemistry and Western Blot.

### Protein isolation and Western blot analysis

Cells from *in vitro* experiments and tumor tissues from *in vivo* experiments were harvested and homogenized in lysis buffer on ice for 30 min. Cell and tissue homogenates were then centrifuged for 15 min at 13,000 RPM at 4 °C. Supernatant was collected with isolated protein, and a Bradford assay was performed to standardize protein concentrations for Western blot and ELISA^35^.

For Western blot analysis, 20 ug protein of each sample was run on a 10% SDS-PAGE gel, transferred to a membrane, and blocked with 5% powdered milk in PBS for 1 h at room temperature. Then, the membranes were incubated with designated antibodies in blocking buffer overnight at 4 °C. The next day, the membranes were washed, appropriate secondary antibodies were applied for 1 h at room temperature, and bands were visualized with an enhanced chemiluminescence kit (Amersham Biosciences, Beijing, China).

### Histopathology and immunohistochemistry

To assess tumor histopathology and smooth muscle actin (SMA) expression, tumor tissues were sectioned and stained with hematoxylin/eosin staining and immunohistochemistry (IHC) was performed. For IHC, tissue sections were rinsed in PBS, blocked with 10% nomal goat serum in PBS for 1 h at room temperature, and incubated overnight with rabbit anti-α-SMA antibody at the manufacturer’s recommended dilution (Abcam, Cambrige, MA, USA). The next day, the tissue sections were washed and incubated in peroxidase-labeled goat anti-rabbit secondary antibodies (Dako Cytomation, Copenhagen, Denmark). Laser confocal fluorescence microscopy (OLYMPUS A, FV1000) was performed after serial dehydration and mounting of the tissue slides.

### Statistical analysis

Data were presented as means ± SEM. Statistical analysis was performed by using SPSS 19.0 software. For continuous variables with a normal distribution, data was analyzed by one-way ANOVA and unpaired or Mann-Whitney *t*-test. Continuous variables without normal distribution were analyzed by using the Wilcoxon-Rank test. *p* < 0.05 was considered statistically significant.

## Electronic supplementary material

Supplementary material
