# Supplementary material for: Targeted blockade of TGF-β and IL-6/JAK2/STAT3 pathways inhibits lung cancer growth promoted by bone marrow-derived myofibroblasts
Source: Sci Rep. 2017 Aug 17;7:8660. doi: 10.1038/s41598-017-09020-8 (PMC5561133; doi:10.1038/s41598-017-09020-8)
Supplement: Supplementary file 1 — Supplementary material [file 41598_2017_9020_MOESM1_ESM.docx]

**Targeted blockade of TGF-β and IL-6/JAK2/STAT3 pathways inhibits lung cancer growth promoted by bone marrow-derived myofibroblasts**

Jindong Shi^1*^, Jingjing Feng^1^, Juan Xie^1^, Zhoufang Mei^1^, Tianyun Shi^1^, Shengmei Wang^1^, Yong Du^1^, Gong Yang^2^, Yougen Wu^2^, Xiaojiao Cheng^3,4^, Shanqun Li^5^, Liming Zhu^6^, Chung S. Yang^6^, Shuiping Tu^3,4*^ & Zhijun Jie^1*^

^1^Department of Respiratory Medicine, ^2^Department of Central Laboratory, The Fifth People's Hospital of Shanghai, Fudan University, Shanghai 200240, China. ^3^Department of Gastroenterology, Ruijin Hospital, Shanghai Jiaotong University School of Medicine, Shanghai 200025, China. ^4^Department of Gastroenterology, Ruijin Hospital North, Shanghai Jiaotong University School of Medicine, Shanghai 201821, China. ^5^Departments of Respiratory Medicine, Zhongshan Hospital, Fudan University, Shanghai 200032, China. ^6^Department of Chemical Biology, Ernest Mario School of Pharmacy, Rutgers, The State University of New Jersey, Piscataway, NJ 08854, USA. *These authors contributed equally to this work. Correspondence and requests for materials should be addressed to Z. J. (email: [jiezjlxh@163.com](mailto:jiezjlxh@163.com)) or S. T. (email: [tushuiping@yahoo.com](mailto:tushuiping@yahoo.com))

**Supplementary material**


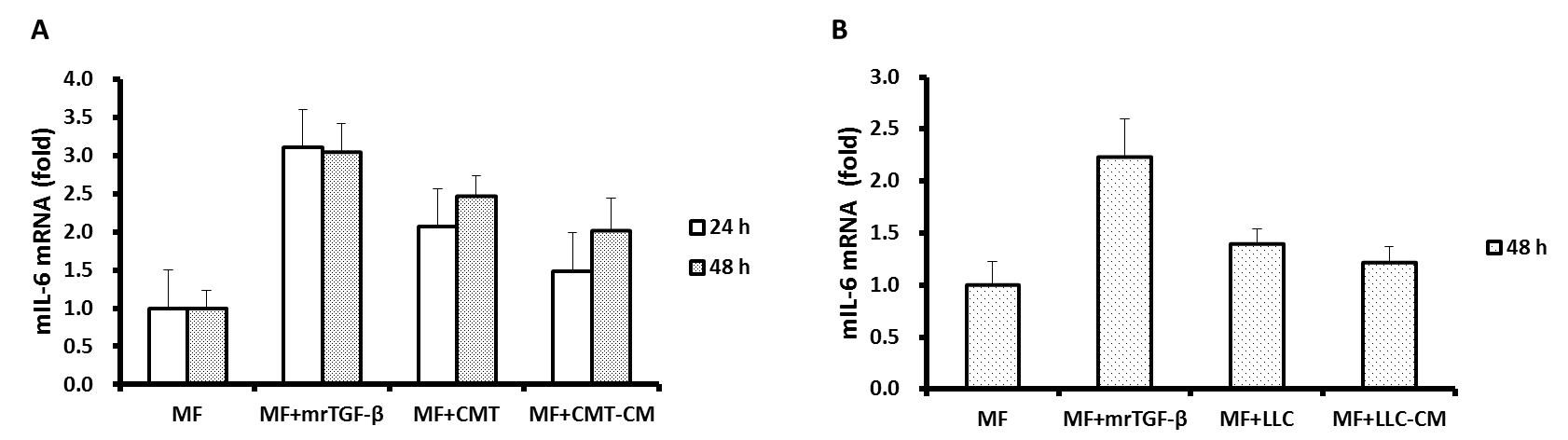


**Supplemental figure 1. Lung cancer cells promote MFs to express IL-6 via TGF-β.** MFs were cultured alone, with mrTGF-β (2 ng/mL), with caner conditioned medium (cancer-CM) or co-cultured with CMT/LLC cells at 24 h and 48 h. mIL-6 mRNA levels in MFs was measured by RT-PCR (Supplemental figure 1A & B). mrTGF-β, lung cancer cells and cancer-CM enhanced expression of mIL-6 in MFs.


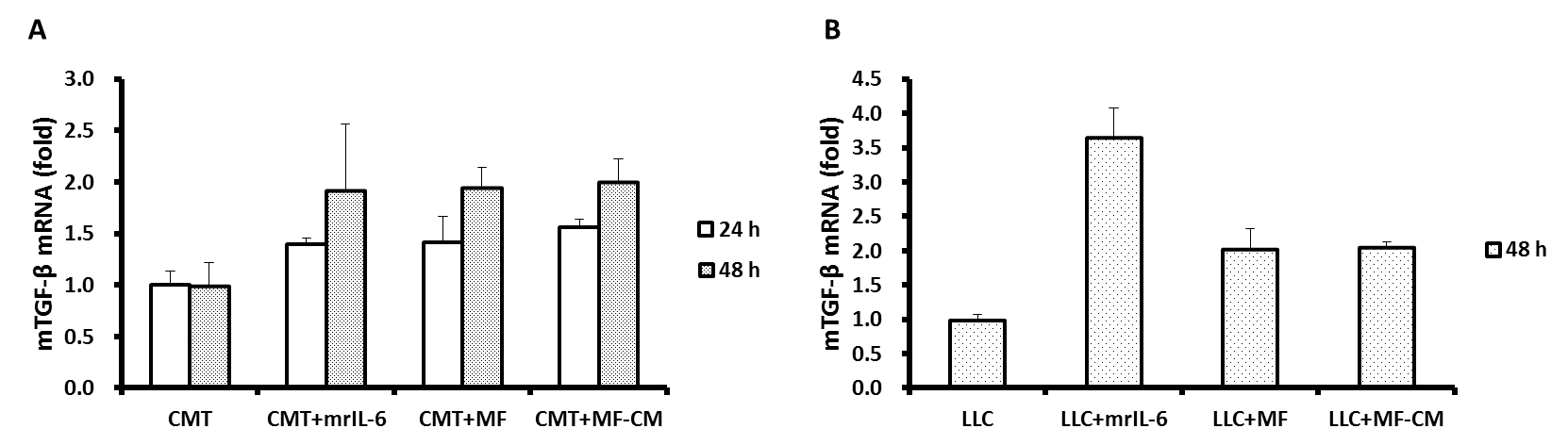


**Supplemental figure 2. MFs promote lung cancer cell to express mTGF-β via IL-6.** CMT/LLC cells were cultured alone, with mrIL-6 (20 ng/mL) or with MF-CM, or co-cultured with MFs at 24 h and 48 h. mTGF-β mRNA levels in MFs in CMT/LLC cells were measured by RT-PCR (Supplemental figure 2A & B). mrIL-6, MFs and MF-CM were both able to enhance the mTGF-β expression in lung cancer cells.





**Supplemental figure 3. SB-431542 and JSI-124 inhibite the proliferation of MFs and CMT/LLC cells in co-culture system.** MFs or CMT/LLCs were cultured in normal medium. Cell proliferation was measured by CCK-8 at 0 h, 24 h and 48h. SB-431542 (10 μM), JSI-124 (0.2 μM), or SB-431542 (5 μM) + JSI-124 (0.1 μM) inhibited MFs proliferation in normal medium (Supplemental figure 3 A). SB-431542 (10 μM), JSI-124 (0.2 μM), or SB-431542 (5 μM) + JSI-124 (0.1 μM) inhibited CMT/LLC cell proliferation in normal medium (Supplemental figure 3B & C).


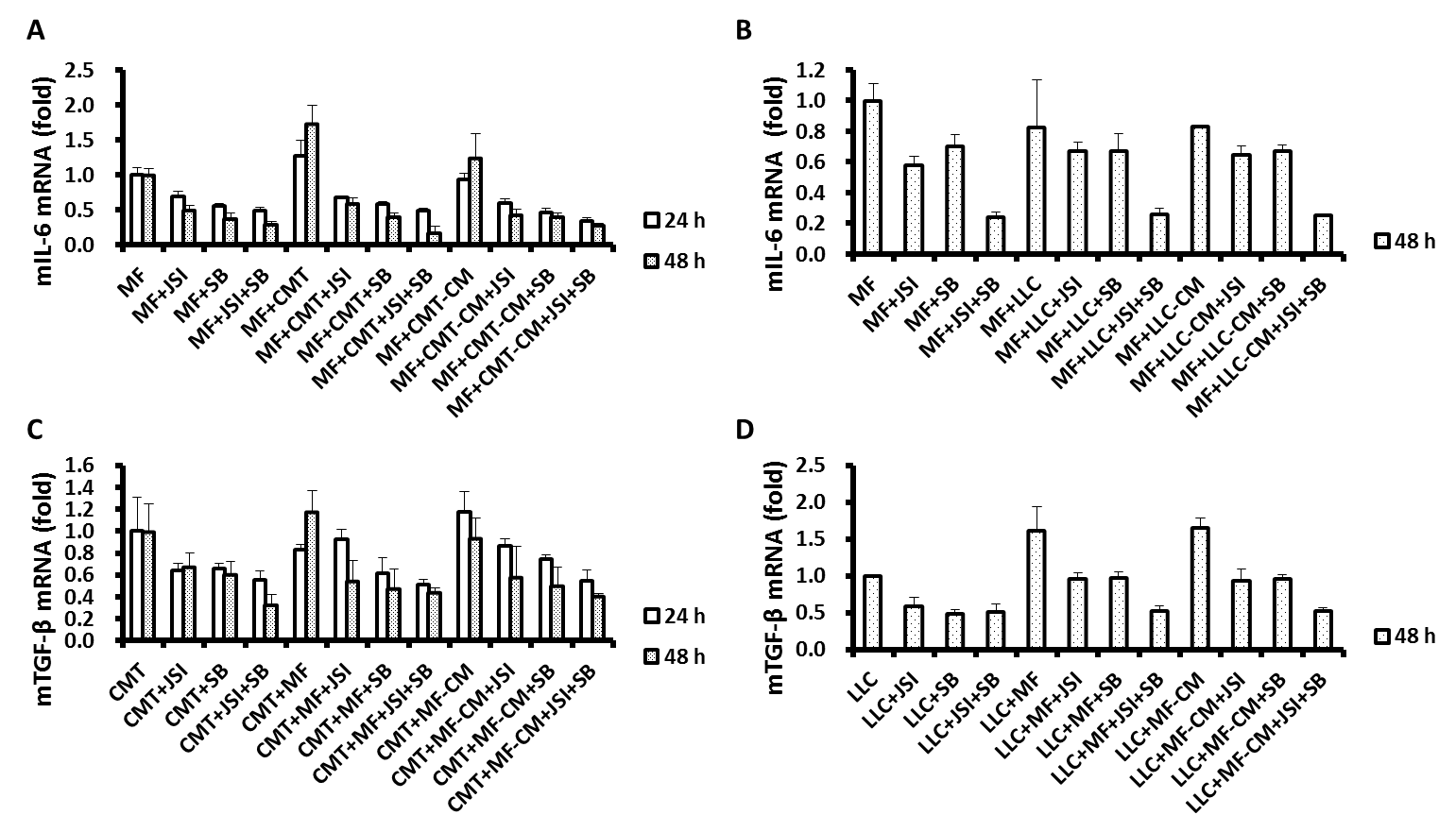


**Supplemental figure 4. SB-431542 and JSI-124 inhibite expression of IL-6 by MFs and expression of TGF-β by lung cancer cells.** MFs (5×10^4^/well) or CMT/LLC cells (5×10^4^/well) were cultured in 6-well plates; In co-culturing, CMT/LLC cells were loaded on the upper chamber and MFs were loaded on the lower chamber of a transwell system. SB-431542 (10 μM), JSI-124 (0.2 μM), or SB-431542 (5 μM) + JSI-124 (0.1 μM) blocked mIL-6 mRNA expressionin of MFs and mTGF-β mRNA expressionin of CMT/LLC cells as determined by RT-PCR (Supplemental figure 4A-D).


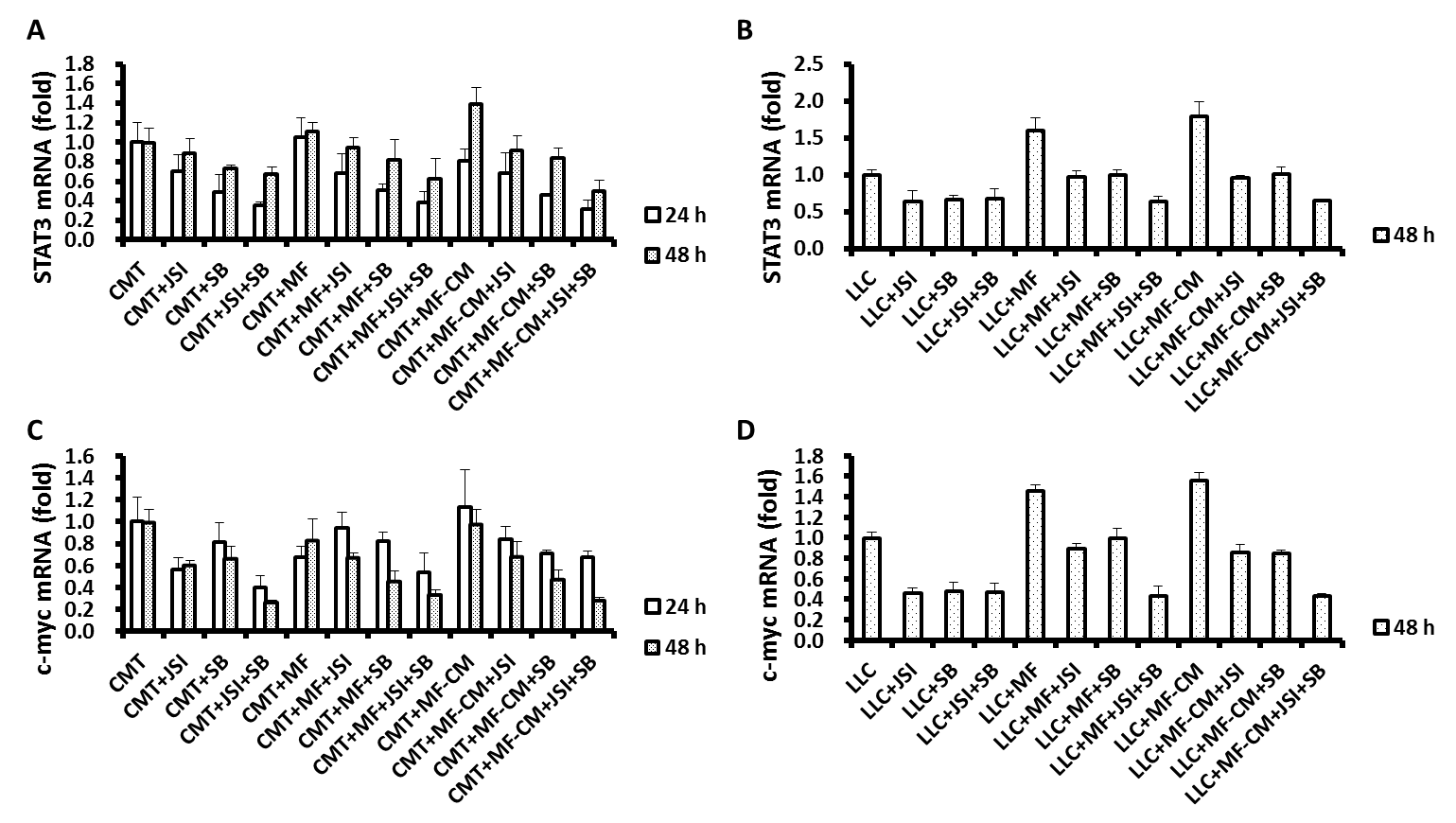


**Supplemental figure 5. SB431245 and JSI-124 decrease the gene expression of STAT3 and c-myc in lung cancer cells.** CMT/LLC cells were cultured in 6-well plates. CMT/LLC cells and MFs were co-cultured in transwell plates. SB-431542 (10 μM), JSI-124 (0.2 μM), or SB-431542 (5 μM) + JSI-124 (0.1 μM) inhibited expression of STAT3 and c-myc genes in lung cancer cells in normal medium and MF-CM (Supplemental figure 5A-D).
